# The Nrf2/HO‐1 pathway participates in the antiapoptotic and anti‐inflammatory effects of platelet‐rich plasma in the treatment of osteoarthritis

**DOI:** 10.1002/iid3.1169

**Published:** 2024-06-11

**Authors:** Guangyu Du, Xuegang Sun, Shengwei He, Lidong Mi

**Affiliations:** ^1^ Department of Bone Surgery The Second Affiliated Hospital of Dalian Medical University Dalian China

**Keywords:** apoptosis, HO‐1, inflammation, Nrf2, osteoarthritis, platelet‐rich plasma

## Abstract

**Introduction:**

We aimed to explore the molecular mechanisms through which platelet‐rich plasma (PRP) attenuates osteoarthritis (OA)‐induced pain, apoptosis, and inflammation.

**Methods:**

An in vivo model of OA was established by injuring rats using the anterior cruciate ligament transection method, whereas an in vitro model was generated by exposing chondrocytes to interleukin (IL)‐1β. Both models were then treated with PRP.

**Results:**

In both the in vivo and in vitro models, OA led to the suppression of the nuclear factor erythroid 2‐related factor 2 (Nrf2)/heme oxygenase‐1 (HO‐1) pathway, whereas treatment with PRP reactivated this molecular axis. Inhibition of the Nrf2/HO‐1 pathway using the Nrf2 inhibitor brusatol or through *Nrf2* gene silencing counteracted the effects of PRP in reducing the tenderness and thermal pain thresholds of OA rats. Additionally, PRP reduced the mRNA expression of *IL‐1β*, *IL‐6*, tumor necrosis factor‐alpha (*TNF‐α*), and matrix metallopeptidase 13 (*MMP‐13*) and the protein expression of B‐cell lymphoma 2 (Bcl‐2), Bcl‐2 associated X‐protein (Bax), and caspase‐3. Furthermore, inflammation and apoptosis were induced by brusatol treatment or *Nrf2* silencing. Additionally, in the in vitro model, PRP treatment increased the proliferation of chondrocytes and attenuated their inflammatory response and apoptosis, effects that were abrogated by Nrf2 depletion.

**Conclusions:**

The Nrf2/HO‐1 pathway participates in the PRP‐mediated attenuation of OA development by suppressing inflammation and apoptosis.

## Graphical abstract

Study results identified a new mechanism, that is the role of Nrf2/HO‐1, underlying the protective role of platelet‐rich plasma in cartilage tissue damage in an ACLT‐induced animal model and IL‐1β‐treated chondrocytes. Meanwhile, our data provide valuable recommendations for the development of promising antiarthritic agents that act on Nrf2.

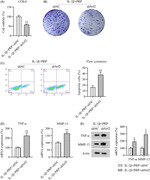

## 1 INTRODUCTION

Osteoarthritis (OA), the most common type of joint disease, results in significant economic losses owing to its high incidence in the general population and the possibility of progressive disability in affected patients.^1^ Current pharmacological treatments for OA only relieve symptoms, such as reducing joint swelling and the severity of pain. Furthermore, no drugs have been clinically shown to elicit chondroprotective effects.^2^

Platelet‐rich plasma (PRP) is a type of autologous blood plasma with above‐baseline levels of platelets and platelet‐derived proteins, including epidermal growth factor (EGF) and transforming growth factor‐beta 1 (TGF‐β1).^3^ The platelets in PRP can secrete a range of mediators and growth factors once activated by exogenous agents.^4^, ^5^ PRP was first used to improve the outcomes of dental implant surgeries, and its musculoskeletal effects have recently gained attention, most notably in the fields of orthopedics and sports medicine.^6^, ^7^, ^8^ Currently, clinical trials are being conducted to confirm whether PRP is capable of easing OA symptoms when administered locally either in the process of surgery (use of PRP gel during orthopedic surgery) or in situ (e.g., at tendinopathy sites or within cartilage or muscle lesions).^9^, ^10^ PRP has also been frequently used to ameliorate OA by facilitating collagen synthesis and chondrocyte proliferation.^11^ It can also suppress extracellular matrix (ECM) degradation and interleukin‐1 beta (IL‐1β)‐induced chondrocyte apoptosis.^12^ However, the molecular mechanisms underlying the effects of PRP remain unclear.

Several studies have shown that the nuclear factor erythroid 2‐related factor 2 (Nrf2)/heme oxygenase‐1 (HO‐1) pathway is involved in OA development.^13^, ^14^, ^15^, ^16^ This pathway has been confirmed to play a critical role in modulating oxidative stress,^17^ inducing apoptosis, and releasing inflammatory mediators.^18^, ^19^ Furthermore, studies have revealed several compounds (including sesamin, glabridin, theaflavin, and lutein) that can activate the Nrf2/HO‐1 pathway and attenuate OA progression.^13^, ^14^, ^15^, ^16^ However, the role of the Nrf2‐associated pathway in the protective effects of PRP against OA progression has rarely been reported.

This study aimed to investigate the effects of PRP on the pathology of OA (especially apoptosis and inflammation) in a rat model of the disease as well as on the proliferation, apoptosis, and inflammatory response of IL‐1β‐stimulated chondrocytes. Additionally, the role of the Nrf2/HO‐1 axis in the PRP‐induced effects was investigated. Brusatol, a quassinoid from the seeds of *Brucea sumatrana*, has been shown to inhibit Nrf2 function. In this study, we used brusatol to inhibit Nrf2 activation. The results provide a theory of the mechanism of action of PRP in OA treatment.

## 2 METHODS

### 2.1 Platelet‐rich plasma preparation

The animal experiments were approved by the Experimental Ethics Committee (The Second Affiliated Hospital of Dalian Medical University; approval number: DY220541). PRP was isolated from 5 mL of blood drawn from Sprague‐Dawley (SD) rats (five males and five females), as previously reported.^20^ The blood specimens were centrifuged for 10 min at 215×*g*, following which the plasma layer above the buffy coat layer was extracted and centrifuged for an additional 10 min at 863×*g*. Then, after discarding the supernatant (without platelets), the remaining liquid (0.3 mL), which was the PRP (1563.9 ± 198.3 × 10^9^/L), was stored at −80°C for the experiments. In total, 3–4 mL of PRP was isolated and collected from the 10 rats. The whole blood sample was analyzed using an XS‐800i hematology analyzer to determine the leukocyte and platelet concentrations. The PRP used in this study contained approximately 1 × 10^8^ leukocytes/L and 2 × 10^12^ platelets/L.

### 2.2 Chondrocyte separation and cultivation

Primary chondrocytes were separated from the articular cartilage of rats and cultivated using previously described methods.^21^ After cleaning and chipping, the rat knee joints were digested with 0.2% type II collagenase (Sigma‐Aldrich) and 0.25% trypsin (Invitrogen) for 2−3 h. Then, the cells were centrifuged (1000×*g* for 8 min at 37°C) and resuspended in 5 mL of Dulbecco's modified Eagle's medium supplemented with 10% fetal bovine serum. Next, chondrocyte growth was determined using an inverted phase‐contrast microscope (CKX31, Olympus). Toluidine blue was used to stain the proteoglycans for chondrocyte identification and counting. The chondrocytes were incubated at 37°C under 5% CO_2_ for 2 days, and cells at passages 3−6 were chosen for the subsequent studies.

### 2.3 Lenviral‐shRNA production

The negative control short hairpin RNA (shRNA‐NC: 5′‐UCA CAA CCU CCU AGA AAG AGU AGA‐3′) and *Nrf2*‐targeting shRNA (shRNA‐Nrf2: 5′‐UCC CGU UUG UAG AUG ACA A‐3′) sequences were provided by Shanghai GenePharma Co. Ltd. After phosphorylation and annealing, the oligonucleotides were cloned into the pLVX‐puro vector and designated as pLVX‐shRNA‐NC and pLVX‐shRNA‐Nrf2, respectively. Lentiviral (LV)‐shRNA‐NC and LV‐shRNA‐Nrf2 particles were produced by means of the triple transfection of 293 T cells (Invitrogen) with the vectors pLVX‐miR‐361 and pLVX‐NC, respectively, along with psPAX2 and pMD2.G. The LV particles were collected from the supernatant after centrifugation of the cell culture medium at 2000 rpm for 10 min.

### 2.4 Osteoarthritis model establishment

Forty‐two SD rats (Vital River Biotech) were randomly classified into seven groups (*n* = 6/group)as follows: control group, OA group (OA rats injected with 50 μL of normal saline), OA + PRP group (OA rats injected with 50 μL of PRP), OA + PRP + Vehicle group (OA rats injected with 50 μL of PRP and orally administered vehicle daily), OA + PRP + brusatol (OA rats injected with 50 μL of PRP and orally administered brusatol daily), OA + PRP + LV‐shNC (OA rats injected with 50 μL of PRP, then infected with LV‐shRNA‐NC particles for 60 h, and finally injected with 50 μL of PRP^22^, ^23^), and OA + PRP + LV‐shNrf2 group (OA rats injected with 50 μL of PRP, then infected with LV‐shRNA‐Nrf2 particles for 60 h, and finally injected with 50 μL of PRP). OA was induced through anterior cruciate ligament transection (ACLT), as previously described.^24^, ^25^ Rats in the control group underwent arthrotomy alone (sham surgery). In brief, the rats in the OA groups were confined to a supine position on the operation table. A 1 cm incision on the medial side of the right knee joint was made to display the medial collateral ligament, which was then removed. The joint capsule was opened to observe the presence of the primary lesion in the cavity. Subsequently, the anterior cruciate ligament was transected, and the medial meniscus was completely removed. The incision was stitched layer‐by‐layer, patched under antiseptic conditions, and fixed. Ampicillin sodium was administered to prevent infection the following day. For LV infection, the LV suspension (5 × 10^7^ PFU/mL) was orthotopically injected into the knee joint at approximately 4 weeks post‐ACLT surgery. After 60 h, PRP was injected into the knee every week for 4 weeks. At 24 h after the final administration, the rats were killed by means of an intraperitoneal injection of pentobarbital sodium, and their knee joints were harvested for subsequent study.

### 2.5 Histological analysis

The rat knee joints were fixed in 4% paraformaldehyde for 48 h, then decalcified in a 10% ethylenediamine tetraacetic acid (EDTA) solution for 4 weeks, and subsequently dehydrated with an ethanol solution. Next, the tissues were embedded in paraffin, and the block was then cut into thin sections (5 μm). Subsequently, the sections were stained with hematoxylin and eosin (H&E) or Safranin‐O for histological assessment. Images of the stained sections were obtained under an inverted microscope (Model IX71, Olympus). On the basis of the Safranin‐O staining results and the Osteoarthritis Research Society International (OARSI; hereinafter OS) ^26^ and Mankin (hereinafter MK) scoring systems,^27^ the extent of cartilage degeneration was determined, as described previously.

### 2.6 Osteoarthritis scoring

The joints were scored by two blinded investigators using the revised MK score, where five regions were scored according to the standardized 14‐point MK scale^28^: the patella, medial and lateral femoral condyles, and tibial plateau. The subchondral bone and synovium were subsequently evaluated using a 5‐ and 4‐point grade, respectively, which were based on the rat‐specific OS metric.^29^ The final scores were evaluated through the addition of the five region‐specific MK scores and the two corresponding OS scores.

### 2.7 Tenderness threshold measurement

The rat tenderness threshold was measured 4 weeks after ACLT, using an electronic YLS‐3E instrument. In this process, the rats were kept in a fixed barrel to render them motionless and comfortable. Then, the hind feet were held down using the flat YLS‐3E head. The tenderness threshold (pressure, g) refers to the pressure measured when the rats scrabble or whine from pain.

### 2.8 Thermal pain threshold measurement

A one‐fourth day gap was allowed between the tenderness threshold and thermal pain threshold assessments. A plantar test instrument was used for the thermal pain threshold measurement. After the rats had stopped their combing and explorative activities in a transparent plexiglass box placed at ambient temperature, infrared light was irradiated onto the hind feet through a glass plate. The thermal pain threshold (m:s) refers to the time point when the rats moved their legs to avoid heat, which was determined in parallel three times every 5−6 min to obtain an average value.

### 2.9 Terminal deoxynucleotidyl transferase‐dUTP nick‐end labeling staining

Tissue sections were fixed in 4% paraformaldehyde for 30 min, following which terminal deoxynucleotidyl transferase‐dUTP nick‐end labeling (TUNEL) staining was performed. The cells were treated with TUNEL reagent (Roche) according to the manufacturer's protocol. Finally, the apoptotic cells were detected and analyzed using a microscope (Nikon). The apoptotic cells showed a green color, while the nuclei were stained blue.

### 2.10 Cell transfection

Chondrocytes were transfected with shRNA‐NC or shRNA‐Nrf2 for 1 d using Lipofectamine 3000 reagent (Invitrogen) and then activated through cultivation with 5 ng/mL IL‐1β (Sigma‐Aldrich) for 1 day. The culture supernatant was harvested, and chondrocyte gene expression was determined by quantification of the isolated total RNA and protein.

### 2.11 Subcellular fractionation

Chondrocytes (3 × 10^6^) were seeded into 6 cm dishes and grown overnight. The cells were then harvested by scraping into 500 μL of 4‐(2‐hydroxyethyl)‐1‐piperazineethanesulfonic acid (HEPES) buffer containing 10 mM HEPES (pH 7.4), 10 mM NaCl, 1 mM KH_2_PO_4_, 5 mM NaHCO_3_, 1 mM CaCl_2_, 0.5 mM MgCl_2_, and 5 mM EDTA with a complete protease inhibitor cocktail. The cells were allowed to swell for 5 min and then disrupted through 50 strokes in a dounce homogenizer. The disrupted cells were centrifuged at 7500 rpm for 5 min, generating a pellet containing nuclei and debris and a supernatant of cytosol and plasma. The pellet was resuspended in 1 mL of Tris buffer, comprising 10 mM Tris (pH 7.5), 300 mM sucrose, 1 mM EDTA, and 0.1% NP‐40, with a complete protease inhibitor cocktail. Then, the suspension was centrifuged again, and the pellet was resuspended and washed twice with Tris buffer. The final pellet contained pure nuclei.

### 2.12 Western blot analysis

Cells and tissues were lysed in a radioimmunoprecipitation buffer containing protease inhibitors, after which the extracted proteins were quantified using a bicinchoninic acid kit (Thermo Fisher Scientific). Eight μg proteins were loaded in each well. Then, the proteins were separated by means of sodium dodecyl sulfate‐polyacrylamide gel electrophoresis (12% gel) and transferred to polyvinylidene difluoride membranes (0.45 µm pore size). Subsequently, the membranes were blocked with 5% bovine serum albumin (Sangon) for 60 min and then incubated overnight with the respective primary antibodies at 4°C. The primary antibodies (all purchased from Abcam) were anti‐p‐Nrf2 (1:500 dilution, Catalog number ab76026), anti‐Nrf2 (1:1000, ab137550), anti‐HO‐1 (1:2000, ab13248), anti‐actin (1:5000, ab8227), anti‐lamin B1 (1:1000, ab65986), anti‐IL‐1β (1:1000, ab9722), anti‐IL‐6 (1:1000, ab6672, Abcam), antitumor necrosis factor‐alpha (TNF‐α) (1:1000, ab6671), anti‐matrix metallopeptidase 13 (MMP‐13) (1:1000, ab84594), anti‐B‐cell lymphoma‐2 (Bcl‐2) (1:5000, ab59348), anti‐Bcl‐2 associated X‐protein (Bax) (1:5000, ab53154), and anti‐cleaved caspase‐3 (1:500, ab2302). The membranes were then washed three times with Tween 20‐containing Tris‐buffered saline and incubated at ambient temperature for 60 min with the secondary antibodies (also from Abcam), which were horseradish peroxidase (HRP)‐conjugated goat anti‐rabbit IgG H&L (1:5000, ab6721) and HRP‐conjugated goat anti‐mouse IgG H&L (1:5000, ab6789). The protein levels were determined using a blot scanner and an ultrasensitive enhanced chemiluminescent substrate (SuperSignal™ West Femto Maximum Sensitivity Substrate Kit, Thermo Fisher Scientific). quantification was performed by ImageJ‐Gel Analysis Software. Data from software calculation was normalized to Control's values in each subgroup.

### 2.13 RNA extraction and quantitative polymerase chain reaction

Total RNA was extracted using the TRIzol reagent (Thermo Fisher Scientific). The quantitative polymerase chain reaction (qPCR) was performed in a 20 µL reaction volume using the following temperature program: 10 min at 95°C, followed by 40 cycles of 15 s at 95°C and 30 s at 60°C, and a final 30 s at 72°C. The following primer sequences were used in this experiment: IL‐1β F: 5′‐CCA CAG ACC TTC CAG GAG AAT G‐3′; IL‐1β R: 5′‐GTG CAG TTC AGT GAT CGT ACA GG‐3′; IL‐6 F: 5′‐AGA CAG CCA CTC ACC TCT TCA G‐3′; IL‐6 R: 5′‐TTC TGC CAG TGC CTC TTT GCT G‐3′; TNF‐α F: 5′‐CTC TTC TGC CTG CTG CAC TTT G‐3′; TNF‐α R: 5′‐ATG GGC TAC AGG CTT GTC ACT C‐3′; MMP‐13 F: 5′‐CCT TGA TGC CAT TAC CAG TCT CC‐3′; and MMP‐13 R: 5′‐AAA CAG CTC CGC ATC AAC CTG C‐3′. Gene transcription was evaluated using SYBR Green staining (Thermo Fisher Scientific). The fluorescence values were standardized using a calibrator, with the glyceraldehyde 3‐phosphate dehydrogenase (*GAPDH*) gene used as the internal reference to obtain the 2^−ΔΔCT^ value (mean of the control samples).

### 2.14 CCK‐8 assay

Cell proliferation was quantified using the Cell Counting Kit‐8 (CCK‐8) assay (Abcam) according to the manufacturer's instructions. In brief, chondrocytes were inoculated into 96‐well plates (30,000 cells/well) and 10 μL of CCK‐8 assay reagent was added. The cells were then incubated for 120 min at 37°C, and the optical density at 450 nm was determined using an M200 auto‐microplate reader.

### 2.15 Colony formation assay

Cells were seeded into 6‐well plates (500 cells/well) and cultivated overnight. Then, the cells were stained with crystal violet according to the manufacturer's protocol and subsequently examined using an Axiovert 200 inverted microscope (Carl Zeiss). The cell colonies were counted using PhotoShop CS6. The experiment was repeated in triplicate.

### 2.16 Flow cytometry

Cell death was assessed using the BD Pharmingen™ FITC Annexin V Apoptosis Detection Kit I with propidium iodide (PI) (556547, BD Biosciences). In brief, after their transfection and resuspension in 20 µL of binding buffer, the cells were treated with 5 µL of PI and 10 μL of annexin V–FITC and then incubated for 20 min in the dark. The number of dead cells was counted using a flow cytometer.

### 2.17 Statistical analysis

The results were analyzed using SPSS Statistics V.11.9. Quantitative data are presented as the mean ± standard error. Student's *t*‐test and one‐way analysis of variance with Tukey's post‐hoc test were used for determining statistical distinctions, with statistical significance set at a *p*‐value below .05.

## 3 RESULTS

### 3.1 PRP alleviated cartilage structural damage and pain

**Figure 1:**
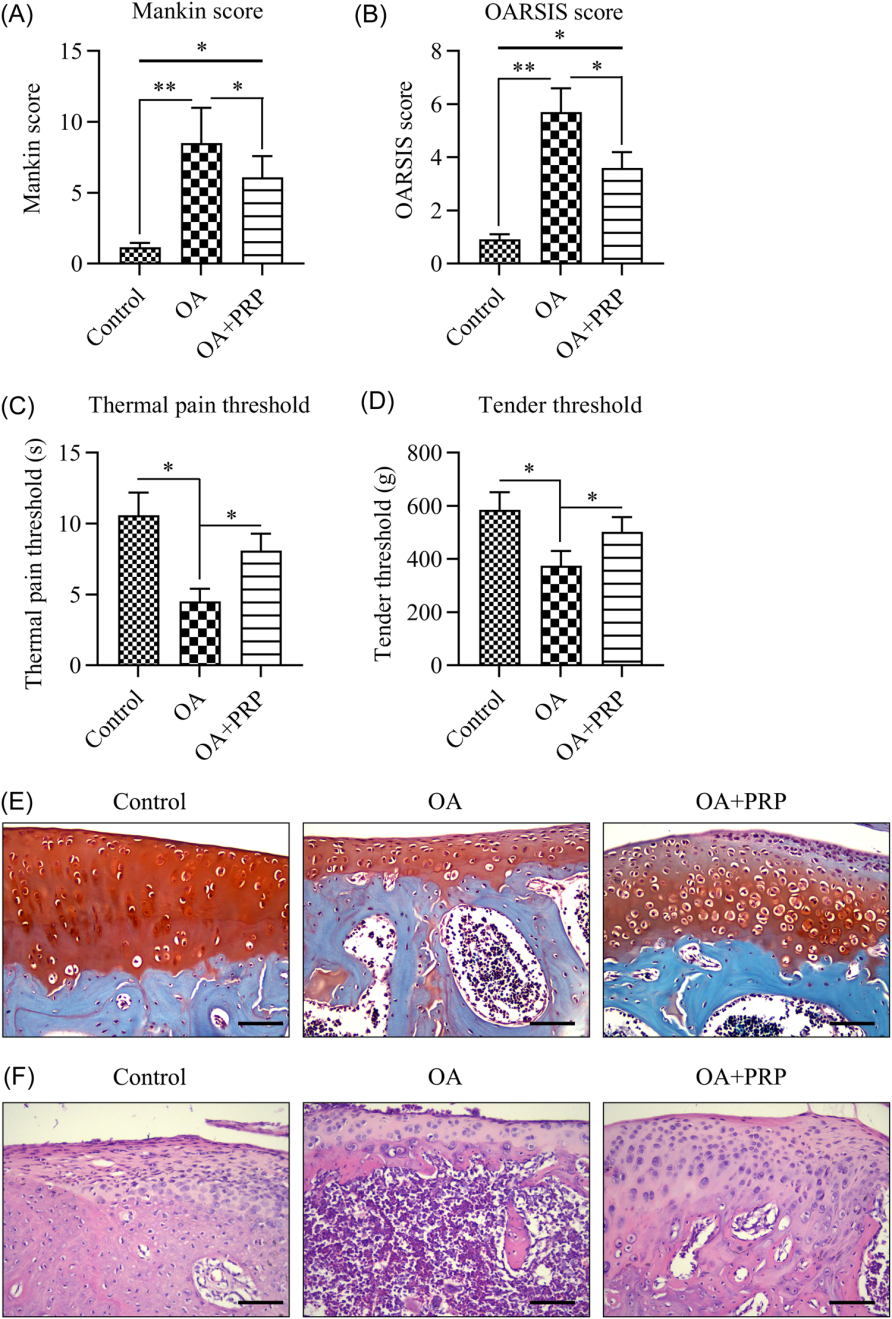
PRP attenuates OA progression in rats. An OA model was established through anterior cruciate ligament transection. Four weeks after modeling, PRP was injected into the knees of the OA rats every week for 4 weeks. (A, B) MK and OS scores. (C, D) TD and TM pain thresholds. (E, F) Histological images of tissue stained with Safranin‐O and H&E (Scale bar, 200 μm). *n* = 6. *p < .05, **p < .01. H&E, hematoxylin and eosin; MK, Mankin; OA, osteoarthritis; OS, Osteoarthritis Research Society International; PRP, platelet‐rich plasma; TD, tenderness; TM, thermal.

To evaluate the protective role of PRP against knee joint disease progression in a rat model of ACLT‐induced OA, MK and OS scores, tenderness and thermal pain thresholds, cartilage histopathology, and OA status were assessed. The OA group showed higher MK (5.7 ± 0.9) and OS scores (8.5 ± 2.5) than the control group (MK, 0.91 ± 0.20; OS, 1.2 ± 0.3), whereas the OA + PRP group (MK, 3.6 ± 0.6; OS, 6.1 ± 1.5) exhibited lower scores than the OA group (Figure 1A,B).

Additionally, both the tenderness and thermal pain thresholds in the OA group were significantly lower than those in the control group. By contrast, the OA + PRP group exhibited markedly higher values for both thresholds than the OA group (Figure 1C,D). These results suggest that PRP treatment alleviates OA‐induced tenderness and pain.

Morphological changes in the cartilage were evaluated using Safranin O and H&E staining after OA modeling. The OA group exhibited more severe cartilage destruction and surface loss relative to the control group. However, PRP reduced cartilage destruction and surface loss (Figure 1E,F), suggesting that its administration could alleviate OA‐induced pathological changes in cartilage tissue.

### 3.2 Activation of the Nrf2/HO‐1 pathway was suppressed in PRP‐treated OA rats

**Figure 2:**
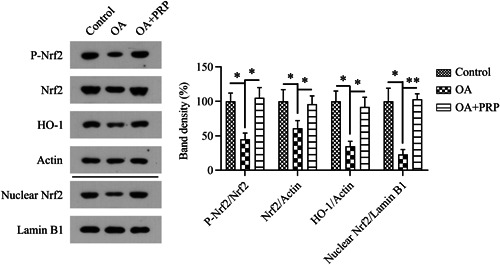
Effects of Nrf2/HO‐1 pathway activation on PRP‐treated OA rat cartilage. Western blot analysis was carried out to determine the protein levels of Nrf2, phosphorylated Nrf2, nuclear‐localized Nrf2, and HO‐1 in homogenates of cartilage tissue from the OA rats. OA, osteoarthritis; PRP, platelet‐rich plasma.

Next, we examined the state of Nrf2/HO‐1 pathway activation in the cartilage tissue of the ACLT‐treated rats. The expression, phosphorylation, and nuclear localization of Nrf2 and HO‐1 were significantly reduced after the induction of OA, whereas PRP treatment markedly upregulated the protein levels of these indicators (Figure 2). Our data suggest that PRP activates the Nrf2/HO‐1 pathway in pathological cartilage tissue.

**Figure 3:**
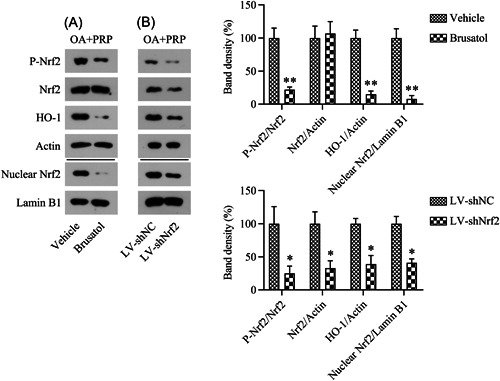
Effects of Nrf2/HO‐1 pathway inhibition and suppression on PRP‐treated OA rat cartilage. The OA model was established through anterior cruciate ligament transection. Four weeks after modeling, PRP was injected into the knees of OA rats every week for 4 weeks. Next, the rats were infected with lentiviral‐shRNA‐NC or lentiviral‐shRNA‐Nrf2 for 3 days and then administrated the vehicle or brusatol by oral gavage for 5 days. Western blot analysis was carried out to determine the protein levels of Nrf2, phosphorylated Nrf2, nuclear‐localized Nrf2, and HO‐1 in homogenates of cartilage tissue from the OA rats. OA, osteoarthritis; PRP, platelet‐rich plasma.

To further determine the role of the Nrf2/HO‐1 pathway in the PRP‐treated OA model, the OA rats were either infected with LV‐shRNA‐Nrf2 to knock down *Nrf2* expression or administered brusatol to block the Nrf2/HO‐1 pathway. Both the knockdown and inhibitor treatments clearly reduced the phosphorylation and nuclear localization of Nrf2 and the level of HO‐1 protein in the cartilage tissue of PRP‐treated OA rats, as determined from the western blot results (Figure 3).

### 3.3 The Nrf2/HO‐1 pathway was found to be associated with the anti‐inflammatory and antiapoptotic effects of PRP on OA rat cartilage tissue

**Figure 4:**
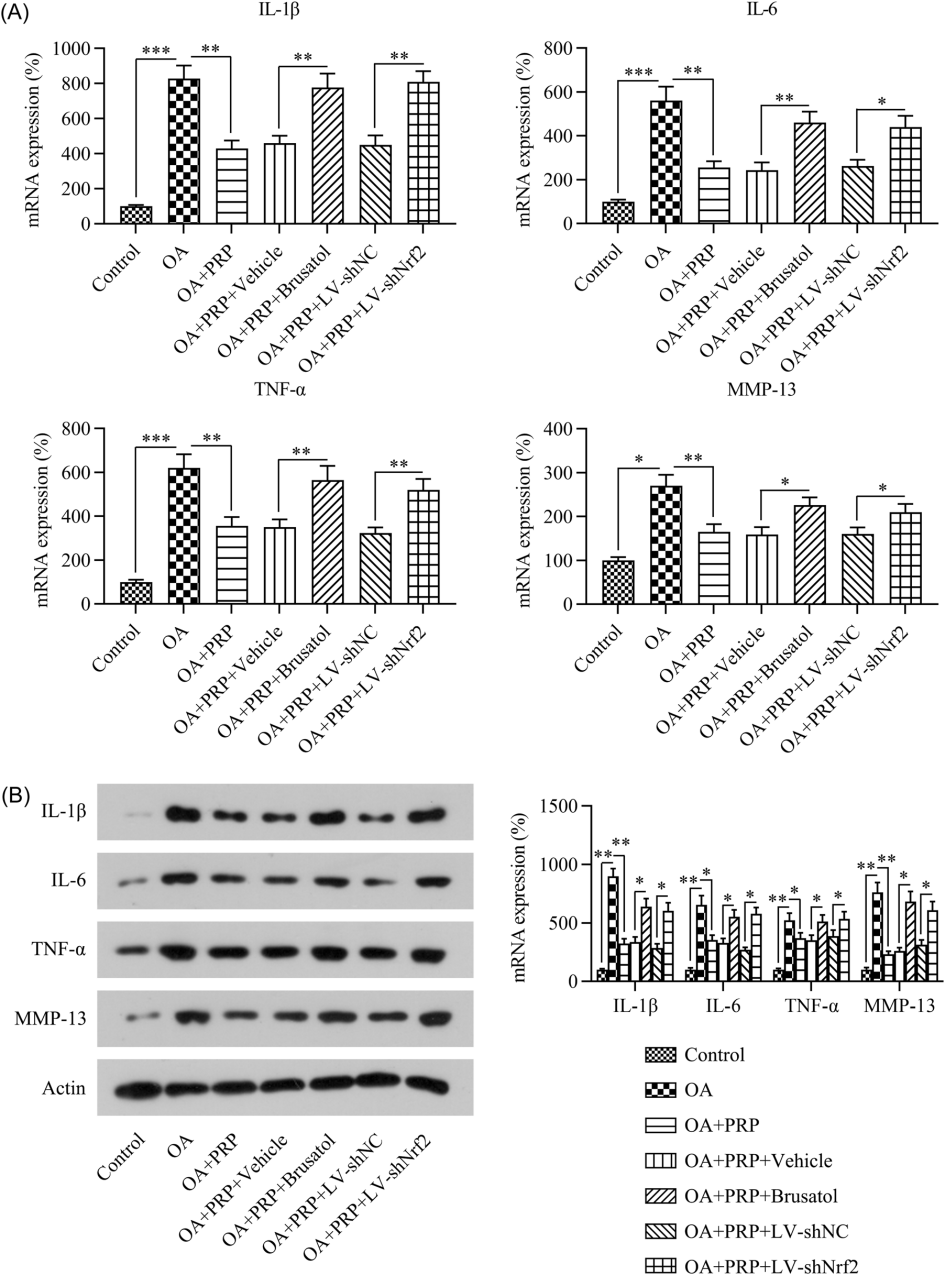
Effects of Nrf2 inhibition on the anti‐inflammatory effect of PRP in OA rats. (A) qPCR and (B) western blot assays of the mRNA and protein levels of inflammatory factors in cartilage tissue. *p < .05, **p < .01, ***p < .001. OA, osteoarthritis; PRP, platelet‐rich plasma.

Both inflammation and apoptosis are associated with cartilage tissue injury and damage during OA development.^30^ Thus, we evaluated whether LV‐shRNA‐Nrf2 silencing or brutasol inhibition of Nrf2 could affect the PRP‐mediated attenuation of osteoarthritic cartilage inflammation and apoptosis. Western blot and qPCR assays were used to respectively measure the protein and mRNA expression levels of IL‐1β, IL‐6, TNF‐α, and MMP‐13. Relative to those in the control group, the mRNA and protein levels of these indicators of inflammation were increased in the OA group. Additionally, PRP treatment reduced the levels of inflammatory factors. Interestingly, the silencing or inhibition of Nrf2 counteracted the influence of PRP on these inflammatory cytokines (Figure 4A,B). These findings indicate that the Nrf2/HO‐1 axis is associated with the PRP‐mediated alleviation of inflammation in osteoarthritic cartilage.

**Figure 5:**
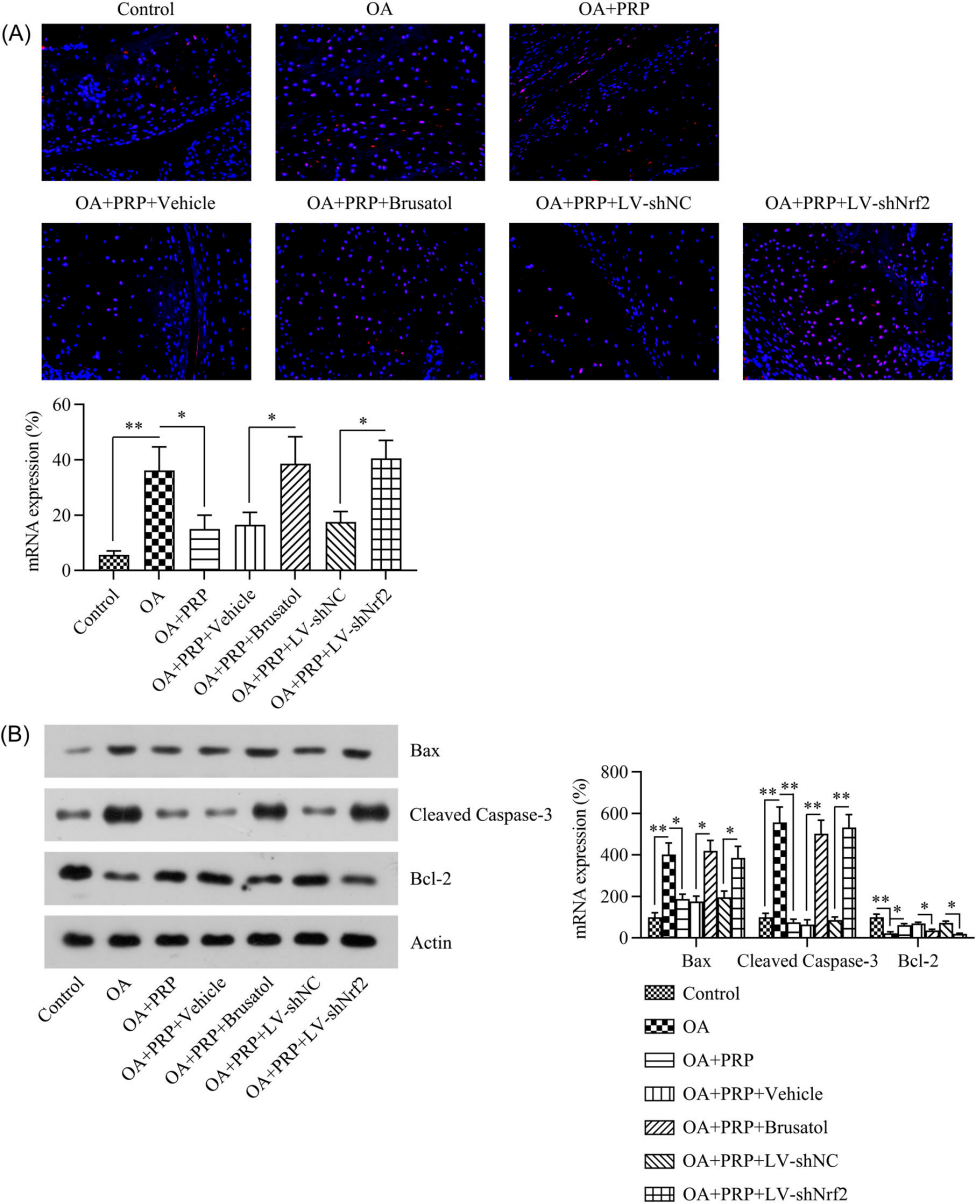
Effect of Nrf2 on the antiapoptotic effect of PRP in OA rats. (A) TUNEL staining assay showing the number of dead cells in the cartilage tissue. (B) Western blot analysis of the Bax, caspase‐3, and Bcl‐2 protein levels in the cartilage tissue.

To assess the various treatment effects on apoptosis, TUNEL and western blot assays were used to, respectively, examine the distribution of dead cells and the protein expression of Bax, Bcl‐2, and caspase‐3. The number of TUNEL‐positive cells in the cartilage tissue was found to be higher in the OA group than in the control group. However, PRP administration decreased the number of TUNEL‐positive cells relative to that in the OA group. Both Nrf2 silencing and inhibition counteracted the effect of PRP on the number of TUNEL‐positive cells (Figure 5A). OA induction caused an increase in the expression of caspase‐3 and Bax protein, and a reduction in that of Bcl‐2 relative to the levels in the control group. However, the PRP‐treated group showed the opposite results. Moreover, Nrf2 silencing or inhibition counteracted the effects of PRP on Bax, caspase‐3, and Bcl‐2 protein levels (Figure 5B). Taken together, these findings indicate that the Nrf2/HO‐1 axis is relevant to the PRP‐mediated decrease in apoptosis in osteoarthritic cartilage.

### 3.4 Nrf2 was responsible for the anti‐inflammatory effect of PRP in IL‐1β‐stimulated chondrocytes

**Figure 6:**
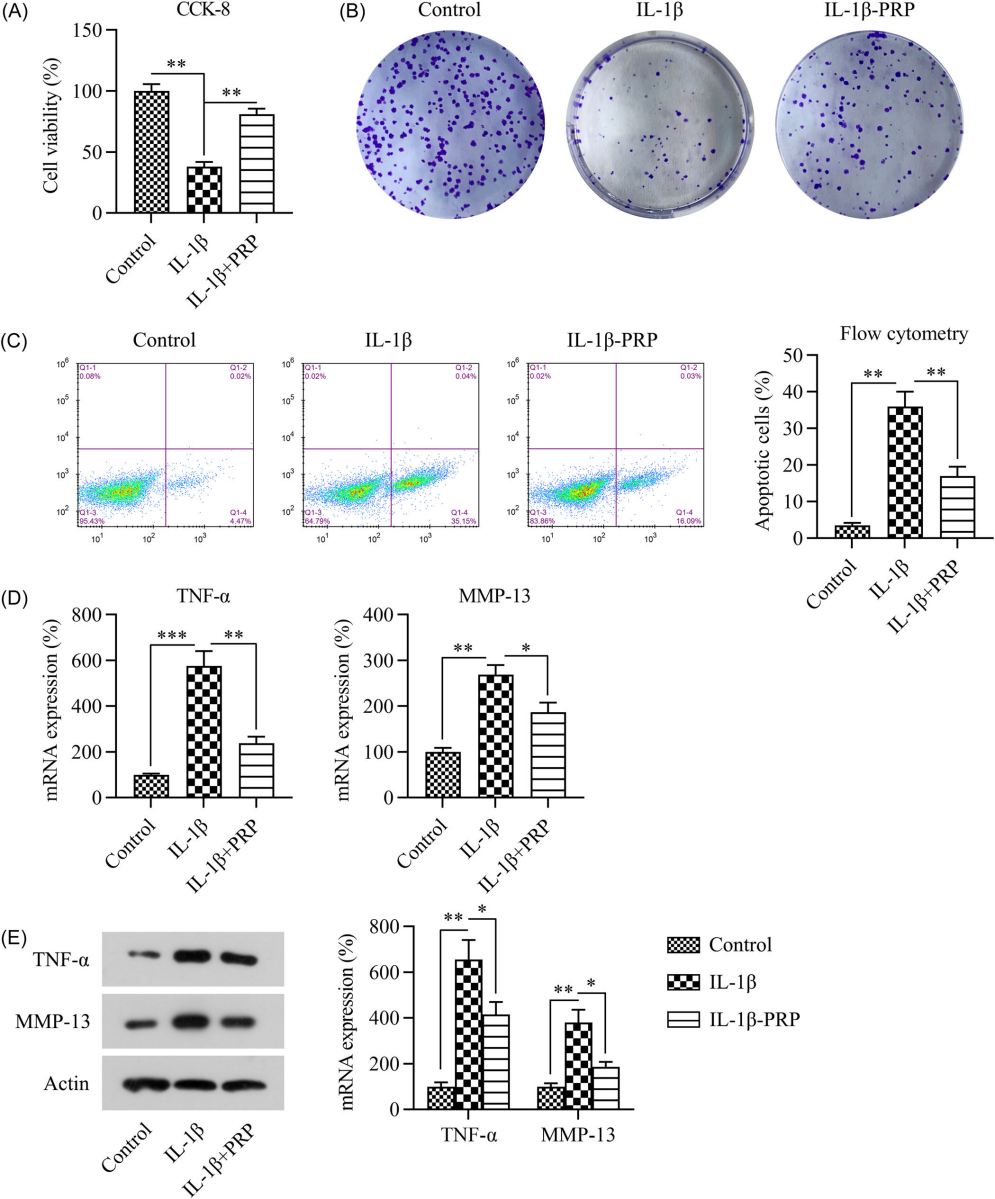
IL‐1β stimulation induces apoptosis and inflammation in rat chondrocytes. The CCK‐8 assay (A) and colony formation assay (B) were used to respectively determine the viability and growth rate of chondrocytes following IL‐1β and PRP treatments. (C) Flow cytometry was used to determine the proportion of apoptotic chondrocytes. (D) qPCR and (E) western blot assays were used to respectively detect the mRNA and protein expression levels of TNF‐α and MMP‐13 in the chondrocytes. *p < .05, **p < .01, ***p < .001. PRP, platelet‐rich plasma.

To establish an in vitro OA cell model, chondrocytes were exposed to 5 ng/mL IL‐1β for 1 d. Then, the CCK‐8 and colony formation assay (CFA) were used to determine the viability and growth rate of the chondrocytes, respectively. IL‐1β treatment reduced the viability of the chondrocytes, whereas PRP treatment recovered the cell survival rate (Figure 6A). These CCK‐8 results were consistent with the CFA data (Figure 6B). The flow cytometry results showed that IL‐1β treatment could induce an increase in chondrocyte apoptosis, whereas PRP decreased the apoptotic cell ratio (Figure 6C). Next, qPCR and western blot assays were used to, respectively, determine the mRNA and protein expression of TNF‐α and MMP‐13. The expression of these two inflammatory indicators was clearly induced by the IL‐1β stimulation and reduced by the PRP treatment (Figure 6D,E). These data suggest that PRP can attenuate IL‐1β‐induced chondrocyte death and inflammation.

**Figure 7:**
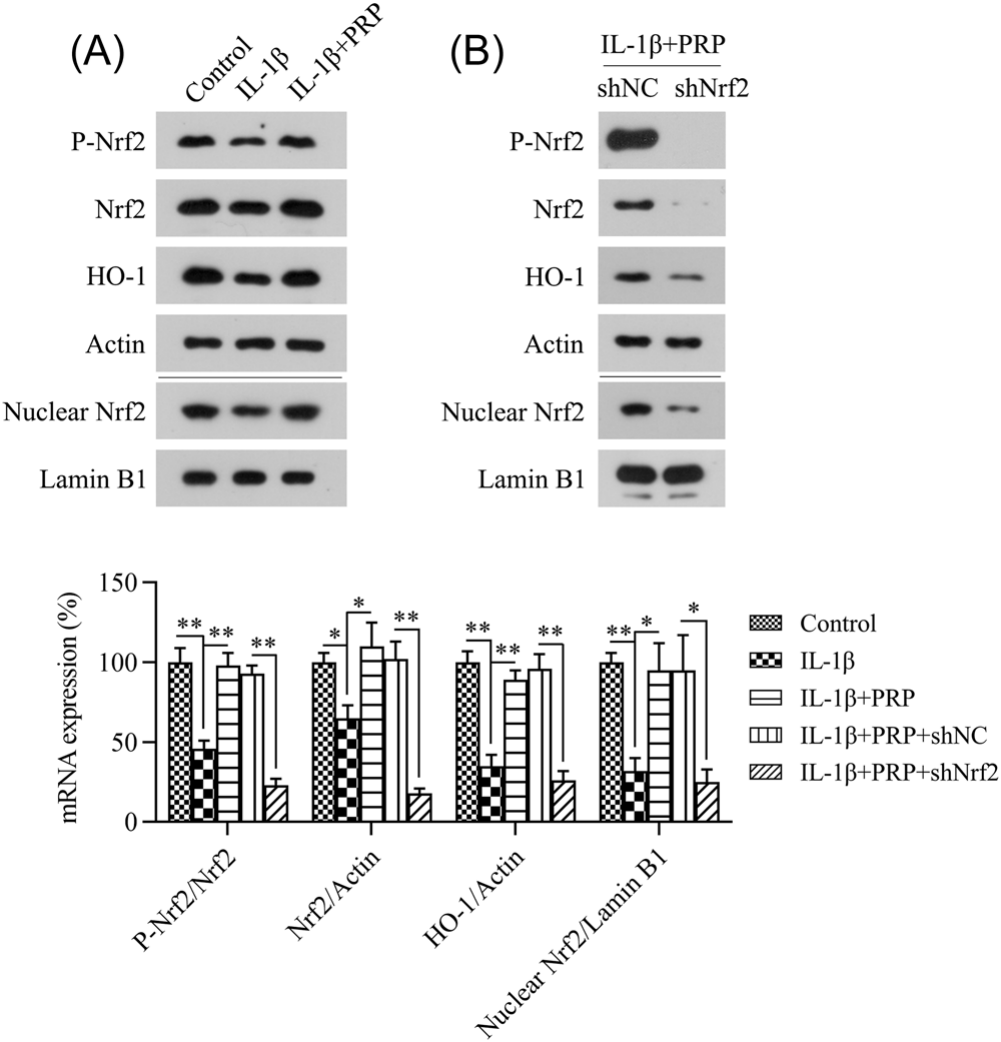
IL‐1β stimulation induces rat chondrocyte apoptosis and inflammation. (A) The cells were treated with 5 ng/mL IL‐1β and 10% PRP for 1 d. Western blot analysis was carried out to determine the protein levels of Nrf2, phosphorylated Nrf2, nuclear‐localized Nrf2, and HO‐1 in the cells. (B) Cells were transfected with shRNA‐NC or shRNA‐Nrf2 for 1 day. Western blot analysis was carried out to determine the protein levels of Nrf2, phosphorylated Nrf2, nuclear‐localized Nrf2, and HO‐1 in the cells. PRP, platelet‐rich plasma.

Western blot analysis was performed to detect the activation of the Nrf2/HO‐1 pathway in IL‐1β‐stimulated chondrocytes. IL‐1β induced the suppression of the Nrf2/HO‐1 pathway, as evidenced by the low Nrf2 and HO‐1 protein levels as well as the reduced phosphorylation and nuclear localization of Nrf2 (Figure 7A).

To elucidate the involvement of Nrf2 and HO‐1 in the PRP‐mediated attenuation of IL‐1β‐induced chondrocyte death and inflammation, we silenced *Nrf2* in IL‐1β‐ and PRP‐treated chondrocytes via transfection with shRNA‐Nrf2. Western blot analysis revealed that *Nrf2* silencing caused the inhibition of Nrf2 and HO‐1 protein expression in the IL‐1β‐ and PRP‐treated chondrocytes (Figure 7B).

**Figure 8:**
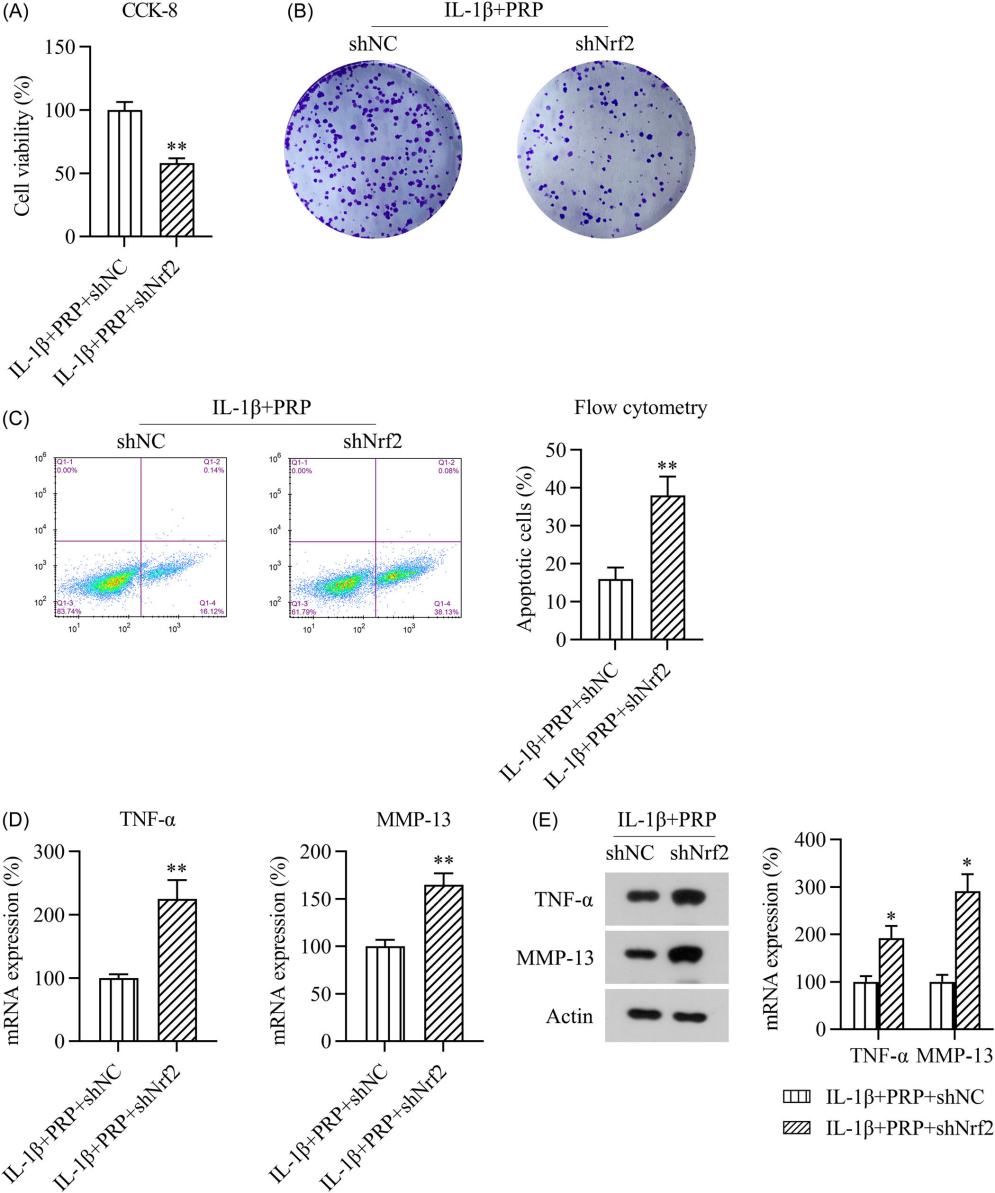
*Nrf2* silencing counteracts the protective effects of PRP against rat chondrocyte apoptosis and inflammation. Cells were transfected with shRNA‐NC or shRNA‐Nrf2 for 1 day and then treated with 5 ng/mL IL‐1β and 10% PRP for 1 day. The CCK‐8 assay (A) and colony formation assay (B) were used to, respectively, determine the viability and growth rate of the treated chondrocytes. (C) Flow cytometry was used to determine the proportion of apoptotic chondrocytes. (D) qPCR and (E) western blot assays were used to respectively detect the mRNA and protein expression levels of TNF‐α and MMP‐13 in the chondrocytes. **p < .01.

Moreover, CCK‐8 staining and CFA were performed a second time to assess the effect of *Nrf2* knockdown on the viability of IL‐1β‐ and PRP‐treated chondrocytes. Both assays indicated that Nrf2 depletion markedly reduced the viability of IL‐1β‐treated chondrocytes, an effect that was reversed by PRP treatment (Figure 8A,B). Flow cytometric analysis also confirmed that *Nrf2* knockdown increased the apoptosis of chondrocytes treated with both IL‐1β and PRP (Figure 8C). Additionally, the mRNA and protein expression levels of TNF‐α and MMP‐13 in the IL‐1β‐ and PRP‐treated chondrocytes increased after *Nrf2* silencing (Figure 8D,E). These data suggest that PRP attenuates apoptosis and inflammation in IL‐1β‐stimulated chondrocytes in an Nrf2‐dependent manner.

## 4 DISCUSSION

OA is a chronic inflammatory disease characterized by progressive cartilage degradation, and joint disability and pain.^31^ Among the diverse methods available to treat the symptoms of OA, PRP has been shown to ease disease‐related pain.^32^ However, the mechanism of action of PRP in attenuating OA pathogenesis remains unknown. This study indicated that PRP could mitigate OA progression by alleviating joint pain, attenuating structural damage to the joint cartilage, and suppressing inflammation and apoptosis in cartilage tissue via activation of the Nrf2/HO‐1 pathway. Moreover, our in vitro experiments showed that PRP treatment restored rat chondrocyte proliferation by suppressing inflammation and apoptosis in the IL‐1β‐stimulated cells.

MK and OS scores are reliable for evaluating the severity of cartilage damage in animal models of ACLT‐induced OA.^27^, ^33^ Xin et al. suggested that PRP treatment could reduce the MK and OS scores.^34^ In this study, we showed that the MK and OS scores of the OA rats, together with the tenderness and thermal pain thresholds, were partially reduced by PRP, indicating that this treatment could attenuate the progression of OA. However, we noticed that the MK and OS scores were not completely restored by PRP administration, suggesting there are other OA factors that this treatment cannot address. Silencing *Nrf2* or blocking Nrf2/HO‐1 pathway activation counteracted the suppressive effects of PRP on the MK and OS scores and tenderness and thermal pain thresholds. These findings suggest that Nrf2 and its downstream molecule, HO‐1 participate in the attenuating effect of PRP on the severity of cartilage damage during OA development.

OA causes cartilage ECM degradation and loss. IL‐1β has been confirmed to play an essential role in cartilage damage by triggering inflammation through its induction of pro‐inflammatory cytokine production.^35^, ^36^ Furthermore, IL‐1β promotes chondrocyte apoptosis by stimulating the expression of Bax and caspase‐3 and reducing that of poly (ADP‐ribose) polymerase (PARP) and Bcl‐2.^37^, ^38^ Additionally, IL‐1β can influence chondrocyte metabolism by repressing ECM protein synthesis and facilitating the release of diverse proteolytic enzymes, including MMPs.^39^, ^40^ These enzymes are an important risk factor that can inhibit type II collagen synthesis.^41^ In particular, MMP‐1, MMP‐3, and MMP‐13 can induce ECM degradation in osteoarthritic articular cartilage.^42^ In our in vitro experiments, we mimicked OA at the cellular level by stimulating chondrocytes with IL‐1β. We found that IL‐1β stimulation reduced cell viability and induced high levels of inflammation and apoptosis in the chondrocytes. Furthermore, incubation of the cells with PRP reversed these IL‐1β‐induced phenotypic changes, which is consistent with the results of a previous study.^43^ Moreover, Nrf2 silencing or inhibition counteracted the influence of PRP on the survival, apoptosis, and inflammation of IL‐1β‐exposed chondrocytes. These data indicate a novel mechanism of OA attenuation by PRP; that is, through the activation of Nrf2.

As a critical transcription factor, Nrf2 is important for protecting cells against oxidative stress damage.^44^ Recent studies have suggested that Nrf2 also has anti‐inflammatory and antiapoptotic effects.^45^, ^46^ Yan et al. proposed that the activation of Nrf2 signaling attenuates OA development by inhibiting the activation of inflammasomes.^47^ Chen et al. found that the suppression of Nrf2/HO‐1 signaling led to increased activation of the NLR family pyrin domain‐containing 3 (NLRP3) inflammasome in OA.^48^ These studies suggest that Nrf2 activation plays an inhibitory role in OA development. To further investigate the anti‐inflammatory and antiapoptotic mechanisms of PRP, its effect on the Nrf2/HO‐1 signaling pathway was investigated. Our results showed that PRP upregulated the expression of both Nrf2 and HO‐1. Furthermore, the inhibitory effects of PRP against chondrocyte inflammation and apoptosis were reduced through the silencing of the *Nrf2* gene and inhibition of its protein activity. These data suggest that the anti‐inflammatory effects of PRP are mediated through the activation of the Nrf2 signaling pathway.

In summary, this study has identified a new mechanism underlying the protective role of PRP against cartilage tissue damage in an animal model of ACLT‐induced OA as well as in attenuating inflammation and apoptosis in IL‐1β‐stimulated chondrocytes; that is, the role of the Nrf2/HO‐1 pathway. However, one limitation of this study is the lack of data from patient samples. The use of patient samples can serve as validation of the efficacy of PRP in treating OA and the role that Nrf2 plays in promoting the therapeutic effects.

## AUTHOR CONTRIBUTIONS

**Guangyu Du**: Conceptualization; formal analysis; methodology; writing—review and editing. **Xuegang Sun**: Conceptualization; formal analysis; investigation. **Shengwei He**: Formal analysis; investigation; validation. **Lidong Mi**: Data curation; formal analysis; methodology; writing—original draft; writing—review and editing.

## CONFLICT OF INTEREST STATEMENT

The authors confirm that there are no conflicts of interest to declare.

## ETHICS STATEMENT

The animal experiments were approved by the Experimental Ethics Committee (The Second Affiliated Hospital of Dalian Medical University; approval number: DY220541).

## Data Availability

All data generated or used during the study appear in the submitted article.
